# Applications of Artificial Intelligence in Gastrointestinal Endoscopic Ultrasound: Current Developments, Limitations and Future Directions

**DOI:** 10.3390/cancers16244196

**Published:** 2024-12-17

**Authors:** Yizhong Wu, Daryl Ramai, Eric R. Smith, Paulo F. Mega, Abdulrahman Qatomah, Marco Spadaccini, Marcello Maida, Apostolis Papaefthymiou

**Affiliations:** 1 Department of Internal Medicine, Baylor Scott & White Round Rock Hospital, Round Rock, TX 78665, USA; eric.smith2@bswhealth.org; 2 Division of Gastroenterology, Hepatology and Endoscopy, Brigham and Women’s Hospital, Boston, MA 02115, USA; 3 Gastrointestinal Endoscopy Unit, Universidade de Sao Paulo Hospital das Clinicas, São Paulo 05403-010, Brazil; 4 Department of Endoscopy, Humanitas Research Hospital, 20089 Rozzano, Italy; marco.spadaccini@humanitas.it; 5 Department of Medicine and Surgery, School of Medicine and Surgery, University of Enna ‘Kore’, 94100 Enna, Italy; marcello.maida@unikore.it; 6 Digestive Diseases and Surgery Institute, Cleveland Clinic, London SW1X 7HY, UK; appapaef@hotmail.com

**Keywords:** artificial intelligence, endoscopic ultrasound, applications, developments, future

## Abstract

Endoscopic ultrasound (EUS) effectively diagnoses malignant and pre-malignant gastrointestinal lesions. In the past few years, artificial intelligence (AI) has shown promising results in enhancing EUS sensitivity and accuracy, particularly for subepithelial lesions (SELs) like gastrointestinal stromal tumors (GISTs). Furthermore, AI models have shown high accuracy in predicting malignancy in gastric GISTs and distinguishing between benign and malignant intraductal papillary mucinous neoplasms (IPMNs). The utility of AI has also been applied to existing and emerging technologies involved in the performance and evaluation of EUS-guided biopsies. These advancements may improve training in EUS, allowing trainees to focus on technical skills and image interpretation. This review evaluates the current state of AI in EUS, covering imaging diagnosis, EUS-guided biopsies, and training advancements. It discusses early feasibility studies and recent developments, while also addressing the limitations and challenges. This article aims to review AI applications to EUS and its applications in clinical practice while addressing pitfalls and challenges.

## Simple Summary

Endoscopic ultrasound (EUS) is widely used to diagnose lesions in the gastrointestinal (GI) tract. Artificial intelligence (AI) holds the potential to enhance the diagnostic capability of diagnostic EUS. While in their experimental phase, several models have been developed to demonstrate the accuracy, sensitivity, and specificity of AI in EUS. These models show the application of AI to facilitate tissue diagnosis and endoscopic training. We provide a comprehensive review of the use of AI in EUS.

## 1. Introduction

Artificial intelligence (AI) is a rapidly growing field with broad applications within gastrointestinal endoscopy [1]. Advanced endoscopists performing endoscopic ultrasound (EUS) stand to benefit from the developments of AI given its potential in imaging analysis. To this end, AI algorithms are trained on extensive image-based datasets.

At the heart of advancements in AI are neural networks, algorithms inspired by the human brain and consisting of interconnected nodes or “neurons” that receive inputs and output information [2]. Artificial neural networks (ANNs) began with simple information flow between nodes. An iterative advancement, convolutional neural networks (CNNs) involve additional algorithmic steps and have superseded earlier ANNs, becoming the most utilized type of neural network [3].

By learning from image sets, neural networks can detect subtle features and abnormalities that might be missed by the human eye. For instance, AI systems have shown remarkable proficiency in identifying tumors, cysts, and other pathological conditions [4]. This capability not only enhances diagnostic precision but also accelerates workflow.

Furthermore, AI can enhance the practice of ultrasound imaging by analyzing and highlighting fleeting, real-time image features and offloading part of the cognitive strain on operators.

This article aims to review AI applications to EUS and its applications in clinical practice. In doing so, readers can better understand its early developments and how the field has developed to the present day. We searched PubMed, Embase, Cochrane, and Google Scholar from inception until October 2024 for studies on AI applications in EUS. These studies generally utilized retrospective imaging data split into a training image set to develop an AI model and a validation image set for testing.

The article will discuss developments in using AI in EUS for the detection and diagnosis of neoplasms, improving the EUS-guided biopsy process and training of new operators. It will then address pitfalls and challenges as well as future directions that may address these current limitations.

## 2. Detection and Diagnosis of Neoplasms

EUS allows for the diagnosis of malignant and pre-malignant GI lesions through direct sonographic visualization. An area where its impact has been demonstrated is the differentiation of different types of lesions with similar features. Sensitivity and specificity of the modality can be very operator and anatomy dependent. AI can augment the abilities of an endoscopist to achieve higher sensitivity and accuracy.

### 2.1. AI in EUS for Subepithelial Lesions

Subepithelial lesions (SELs) include gastrointestinal stromal tumors (GISTs), leiomyomas, lipomas, and neuroendocrine tumors. GISTs are mesenchymal tumors that have malignant potential and are difficult to distinguish from non-GIST tumors using EUS [5]. Table 1 lays out the studies involving the use of AI-enhanced EUS for SELs and summarizes their results.

Minoda et al. showed in their study that their AI had better accuracy, sensitivity, and specificity independent of lesion size when compared against experienced endoscopists. The accuracy, sensitivity, and specificity for SELs smaller than 20 mm were 86.3, 86.3, and 62.5%, respectively, for the AI group. By comparison, these metrics were 73.3%, 68.2%, and 87.5%, respectively, for the human expert group. For SELs larger than 20 mm, the accuracy, sensitivity, and specificity were 90.0%, 91.7%, and 83.3%, respectively, in the AI group. Contrasting with this, EUS experts achieved 53.3, 50.0, and 83.3%. The area under the curve for the diagnostic yield of the AI group was significantly higher (*p* = 0.007) than that of the expert group at 0.965 and 0.684, respectively [5].

Seven et al. utilized a cohort of 55 patients who underwent EUS before surgical resection for gastric GISTs to create an AI model to predict malignancy risk. This totaled 685 images of GISTs that served as the training data set for the AI system. A total of 70% of the generated images were used for AI training while 30% were used to test AI diagnoses. When dividing their data into high-risk and low-risk groups, their model achieved sensitivity, specificity, and accuracy as high as 99.7%, 99.7%, and 99.6%, respectively [6].

**Figure 1:**
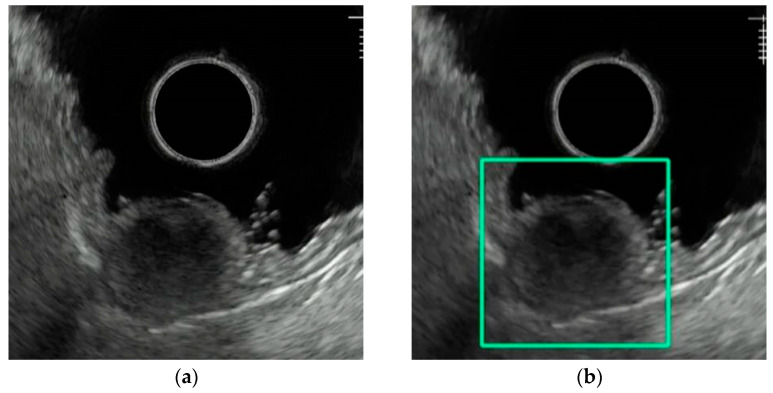
Images of AI in EUS for evaluating GISTs from Hirai et al.: (**a**) EUS image depicting a GIST without AI detection; (**b**) image with AI detection.

Hirai et al. investigated the accuracy of a CNN they developed and found that the AI outperformed endoscopists in multiple domains. The sensitivity and accuracy of the AI system showed their best performance against experienced endoscopists for differentiating GIST/schwannoma from other SELs with sensitivity of 100% vs. 84.4% (*p*  <  0.001), accuracy of 95.1% vs. 82.8% (*p*  =  0.003). The specificity of the AI system was comparable to that of the experts at 76.9%, but higher than that of trainees, whose specificity ranged between 50% and 69.2% [7]. Figure 1 illustrates what their AI detection system looks like in practice.

A Korean group led by Kim et al. trained a CNN using 905 EUS images of pathologically confirmed GISTs as well as benign leiomyomas and schwannomas. This test dataset also underwent human analysis by three experienced endoscopists, as well as three trainees. The CNN ultimately outperformed the human group with sensitivity, specificity, and accuracy at 83.0%, 75.5%, and 79.2%, respectively [8]. More recently, Dong et al. developed a CNN to distinguish SELs from leiomyomas. They utilized retrospective data from 1101 participants with SELs for AI system development. A cohort of 241 participants with SELs were recruited for external validation of their system. Another cohort of 59 participants with SELs was prospectively enrolled to assess the real-time clinical application of the AI system. This study involved more patients than the study by Kim et al. and was able to achieve better performance with sensitivity, specificity, and accuracy of 90.3%, 93.0%, and 91.7%, respectively [9].

**Table 1:** Role of AI in EUS detection and diagnosis of subepithelial lesions.

Study	Country of Study	AI Model	Area of Interest	Number of Training Images	Number of Test Images	Primary Outcome
Minoda 2020 [5]	Japan	CNN	GISTs vs. non-GIST lesions	173	100	Sensitivity 90.0%, Specificity 91.7%, Accuracy 83.3%
Kim 2020 [8]	Korea	CNN	GISTs vs. non-GIST lesions	587	212	Sensitivity 83.0%, Specificity 75.5%, Accuracy 79.2%
Seven 2021 [6]	Turkey	CNN	GISTs vs. non-GIST lesions	685	153	Sensitivity 75.0%, Specificity 73.0%, Accuracy 66.0%
Hirai 2022 [7]	Japan	CNN	GISTs vs. non-GIST lesions	509	122	Sensitivity 98.8%, Specificity 67.6%, Accuracy 89.3%
Dong 2024 [9]	China	CNN	GISTs (SELs) vs. Leiomyoma	1101	241	Sensitivity 90.3%, Specificity 93.0%, Accuracy 91.7%

Abbreviations: CNN (convolutional neural network), GIST (gastrointestinal stromal tumor), SEL (subepithelial lesion).

### 2.2. AI in EUS for Pancreatic Lesions

EUS examination of pancreatic lesions offers high diagnostic yield and can produce high-quality images. However, there are specific areas where EUS falls short, including differentiating between benign and malignant intraductal papillary mucinous neoplasms (IPMNs). A group led by Kuwahara et al. utilized deep learning on EUS images from 50 patients with IPMNs to develop an AI model that can differentiate between benign tumors and malignancy. Their model showed sensitivity, specificity, and accuracy of 95.7%, 92.6%, and 94%, respectively, compared to the 56% accuracy of human operator diagnosis [10].

Saraiva et al. developed a deep learning model using 55,450 EUS images from 149 procedures aimed at analyzing both solid and cystic lesions. Their algorithm was designed to differentiate pancreatic ductal adenocarcinoma (PDAC) from pancreatic neuroendocrine neoplasms (PNENs) and other rarer solid lesions. Their model was also trained on cystic lesions. They were able to identify PDAC with sensitivity, specificity, and accuracy of 99.4%, 98.6%, and 99.3%, respectively. For PNENS, they had similar results with sensitivity, specificity, and accuracy of 97.2%, 99.8%, and 99.4%. Their AI model also showed similar performance when differentiating adenocarcinoma from neuroendocrine tumors, and mucinous from non-mucinous cystic lesions [11].

Bang et al. independently developed their own neural networks to differentiate between cystic and solid pancreatic tumors using data from 202 consecutive patients undergoing EUS of the pancreas. EUS-FNA/B with rapid on-site assessment followed by histology were used as the gold standard for confirming a diagnosis of cystic and/or solid pancreatic lesions. Training was performed using two CNNs, one faster and simpler, and the other slower and more complex. The overall accuracy on cystic pancreatic lesion analysis was 100% for the faster CNN and 91% for the slower CNN. For solid pancreatic masses, accuracy was 100% for the faster CNN and 92% for the slower CNN for solid pancreatic masses, respectively [12].

Marya et al. trained an AI model to differentiate PDACs from atypical autoimmune pancreatitis (A-AIP), a potential radiographic mimic of pancreatic cancer [13]. The model was trained to analyze EUS videos in real time and was able to accurately distinguish A-AIP from PDAC and benign pancreatic diseases such as chronic pancreatitis, as well as from normal pancreas. For differentiating A-AIP and PDAC, the AI model achieved sensitivity and specificity of 90% and 93%, respectively. When distinguishing AIP from chronic pancreatitis, the sensitivity and specificity were 94% and 71%, respectively. Between AIP and normal pancreas, the specificity was 98%. Finally, between A-AIP and non-AIP, the sensitivity and specificity were 90% and 85%, respectively [14].

### 2.3. AI for Cancer Staging with EUS

Beyond applications in EUS for differentiating lesion types, AI systems have also demonstrated efficacy in the staging of malignancy. Uema et al. utilized data from 285 cases of early gastric cancer (EGC) to develop a multi-step AI model for diagnosing invasion depth of EGC. This model was able to achieve similar performance to that of experts with sensitivity, specificity, and accuracy of 66.3%, 88.7%, and 90.4% [15]. This highlights the potential of an AI tool to help operators stage tumors in real time. Table 2 lays out the studies involving the use of AI-enhanced EUS for pancreatic lesions and cancer staging and summarizes their results.

### 2.4. Improving Current Capabilities

AI models trained by discrete groups of investigators have yielded promising results but are limited by the generalizability of each model beyond the population from where their training data is obtained. Researchers in computer science are working on methods such as combo libraries to merge and combine machine learning models, a promising step in developing more generalizable AI models [16]. Optimal training methods for creating CNNs for augmenting the detection and diagnosis of neoplasms via EUS have not been established. Future studies will need to compare the methods used, such as training using a single CNN versus utilizing multiple CNNs of varying complexity.

**Table 2:** Role of AI in EUS detection of pancreatic cancers and cancer staging.

Study	Country of Study	AI Model	Area of Interest	Number of Training Images	Number of Test Images	Primary Outcome
Kuwahara 2019 [10]	Japan	CNN	Malignant vs. benign IPMNs	206	50	Sensitivity 95.7%, Specificity 92.6%, Accuracy 94.0%
Marya 2021 [14]	USA	CNN	AIP vs. neoplasm	460	123	Sensitivity 90.0%, Specificity 93.0%
Sairava 2024 [11]	Portugal, Brazil, USA	CNN	Cystic vs. solid pancreatic lesions	340	38	Sensitivity 99.4%, Specificity 98.6%, Accuracy 99.3%
Bang 2024 [12]	China	CNN	Cystic vs. solid pancreatic lesions	150	52	Accuracy 91–100%
Uema 2024 [15]	Japan	CNN	Early gastric cancer staging	285	135	Sensitivity 66.3%, Specificity 88.7%, Accuracy 90.4%

Abbreviations: CNN (convolutional neural network), IPMN (intraductal papillary mucinous neoplasm), A-AIP (atypical autoimmune pancreatitis).

## 3. Integration into Biopsy

There is heterogeneity between individual EUS operators in terms of the quality of biopsy samples obtained during EUS. AI can support an EUS operator and improve overall consistency and accuracy. This can be specifically achieved by highlighting optimal biopsy locations and depth. As early as 2014, researchers in Japan have been working on integrating AI into the EUS-guided FNA/FNB workflow [17]. While early models were simpler and limited by the types of AI models available at the time, later developments implemented CNNs.

### 3.1. Developments with Rapid On-Site Evaluation

Rapid on-site evaluation (ROSE) involves having the operator, in this case the endoscopist, perform an on-site evaluation of sample adequacy prior to the final read by a cytopathologist. Early on, ROSE-assisted biopsy demonstrated the ability to reduce the number of needle punctures, complication rates, and procedure time, while improving the diagnostic yield when applied to bronchoscopy [18].

Subsequently, ROSE has been shown in numerous studies to be beneficial when paired with EUS and FNB [19,20]. Inoue et al. first studied the feasibility of using AI to assist in performing ROSE. Their machine learning method was effective in assisting on-site visual inspection of cellular tissue in ROSE for EUS-FNA by highlighting areas with a high probability of containing tumor cells [17]. More recent studies have continued to show promising results.

Hashimoto et al. published early results of an AI model applying neural networks to EUS-FNA cytology of PDACs. After training with 500 cytological images, their neural network showed promising results in improving diagnostic performance. Their results showed a diagnostic performance sensitivity, specificity and accuracy of 60% and 69% at the first learning stage, respectively. They achieved sensitivity, specificity, and accuracy of 80%, 80%, and 80%, respectively, at the second learning stage, demonstrating how incremental training of AI can further improve performance [21].

Ishikawa et al. developed an AI-based method for evaluating EUS-FNB specimens in pancreatic diseases using a unique contrastive learning method. Their model was comparable if not slightly superior to macroscopic on-site evaluation (MOSE) performed by EUS experts. Their initial baseline study involved 98 EUS-FNB specimens from 63 patients and standard neural network training, and resulted in an accuracy of 71.8%, lower than the 81.6% of experts [22]. They then utilized contrastive learning, an AI training technique that has demonstrated improvement over standard training [23]. They applied contrastive learning to standard and stained samples of the same image specimen. EUS experts achieved sensitivity, specificity, and accuracy of 88.97%, 53.5%, and 83.24%, respectively, while those of the AI-based diagnostic method using contrastive learning were 90.34%, 53.5%, and 84.39% [22]. This demonstrates an advantage to using this alternative training method in AI-based evaluation for EUS-FNB.

Kuwahara et al. made further advancements in the application of AI to ROSE. They trained a model using images of pancreatic cell clusters from 110 patients between 2019 and 2021. Their AI model showed higher accuracy and faster performance than the diagnostic performance of 21 endoscopists [24]. This built on the group’s earlier work that retrospectively reviewed 85 patients with EUS-FNA specimens and developed an ANN that achieved sensitivity, specificity, and accuracy of AI of 95.7%, 91.9%, and 92.9%, respectively, for differentiating malignant from benign cystic lesions [25].

**Figure 2:**
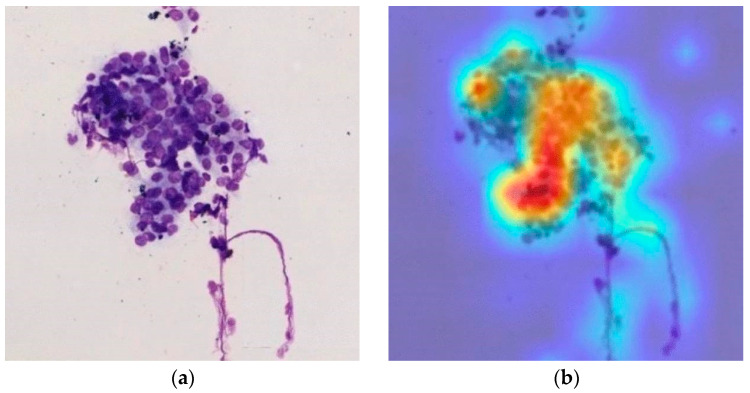
Images for an application of AI to ROSE by Fujii et al.: (**a**) original biopsy image; (**b**) image after evaluation by the AI with heat map representing the AI’s confidence in there being cancer.

Figure 2 shows the results of an AI model created by Fujii et al. to evaluate biopsy slide images obtained via endoscopic ultrasound [26]. They collected 4059 EUS-FNA images from 36 patients with pancreatic cancer. Their AI system achieved sensitivity, specificity, and accuracy of 87.5%, 79.7%, and 83.7%, respectively. They employed several data augmentation techniques and compared techniques, and found that for AI applied to ROSE, a technique called geometric transformation achieved the best result. The investigators do suspect that different techniques may be optimal for different applications.

### 3.2. Other Advancements in AI for EUS Guided Biopsy

Qin et al. were able to develop a hyperspectral imaging (HSI) neural network to assist in EUS-guided FNA pancreatic cytological specimens. HSI is a newer technology capable of visualizing a broader span of the electromagnetic spectrum compared to conventional video imaging, which only visualizes visible light. Their model was able to achieve accuracy of 92.04% with sensitivity and specificity of 93.10% and 91.23%, respectively. This study portends the ability of AI to train and adapt to new technologies [27].

Real-time AI analysis can augment EUS biopsy procedures by identifying the optimal location and depth for biopsy needle insertion. It can also give endoscopists feedback on sample tissue quality. AI tools can also use characteristics of the target lesion such as the diameter and location to assist users in selecting optimal needle size and type [28]. These pioneering studies show that AI can have a positive impact on EUS-guided biopsies. Table 3 lays out the studies involving the integration of AI into EUS guided biopsies and summarizes their results.

## 4. Augmentation of Training

EUS is a skill that requires constant training and practice to become proficient. The traditional approach to medical training has been an apprenticeship model, and training for EUS has been no exception. Trainees learn by observing attendings, and then learn in practice during procedures. Procedure volume built a trainee’s hands on experience, and feedback consisted of subjective assessments. Typically, the depth of training has been measured in the number of procedures, with different guidelines being published for the minimum number of completed procedures required for trainees [29]. Despite most program directors and trainees surveyed believing specific metrics are important, most endoscopy training programs rely on the imperfect apprenticeship model. AI has demonstrated utility in improving the training experience for novices and can be beneficial in improving on current training methods.

Yao et al. performed a study on the effect of AI on colonoscopies performed by novice operators [30]. They compared the lesion detection ability of novices, novices assisted by AI, and experts. The results of their investigation across three hospitals in China showed that AI-assisted novices had significantly lower adenoma miss rates (18.82% vs. 43.69%, *p* < 0.001). They also found that AI-assisted novices met their non-inferiority margin. While not specifically studying the effect of AI in EUS training, this study does presage the utility of AI in training endoscopists.

Several groups in China have developed AI tools specifically designed as training aids in EUS. Contrast-enhanced harmonic EUS (CH-EUS) is a newer technique in EUS that can distinguish gallbladder cancer and pancreatic cancer and has further utility in guiding FNA and biopsy [31]. As a newer technology, it is not widely disseminated, which presents a challenge for training new operators. Tang et al. created a deep-learning-based CH-EUS diagnosis system and evaluated its value in training. They found that the intersection over union, a measure of localization accuracy, of trainees significantly improved from 0.80 to 0.87 (*p* = 0.002). They also found that the average lesion identification time of trainees improved from 22.75 to 17.98 s (*p* < 0.01) [32].

For general EUS training, another group led by Zhang et al. developed a system they dubbed BP MASTER (pancreaticobiliary master) for EUS training. The model was validated, and then a crossover study was performed to evaluate the tool’s effect on trainees. Trainee recognition accuracy improved from 67.2% to 78.4% (*p* < 0.01) using BP MASTER [33]. This tool was further developed into the EUS-intelligent and real-time endoscopy analytical device (EUS-IREAD) device, which was able to reduce the missed scanning rate of EUS stations from 14.3% to 4.5% and missed scanning rate of anatomical structures from 17.4% to 6.2% [34].

Bonmati et al. studied the feasibility of using an AI that could interpret an operator’s vocal input of anatomical structure names to label EUS images. Their model underwent an initial round of training and achieved an accuracy of 76% in labeling EUS images from a dataset including five different labels [35]. The ability to use vocal inputs to automatically tag structures seen on EUS represents an improvement in quality of life for an EUS operator, especially for trainees who are inexperienced in the technical aspects of EUS. However, while quality of life improvements via AI provide support to novices, it has not been objectively demonstrated to result in improvements to the learning curve.

These advances in creating trainee-friendly tools stand to augment the training experience in EUS. AI-augmented training experience in the early stages of training can allow trainees to focus on technical skills while learning to recognize and interpret EUS images. Table 4 lays out the studies involving the use of AI in EUS training and summarizes their results.

## 5. Limitations and Potential Pitfalls

AI holds significant potential in the fields of diagnostic and therapeutic EUS. Despite high degrees of accuracy across multiple fields within EUS, AI also carries bias based on the data used for training and specific algorithms used [36]. An AI model developed by a particular group based on data from their patient population may not be reflective of other localized patient populations. To this end, high-quality data are needed to limit biases and maximize accuracy.

Additionally, the training methods used in the development of an AI model vary between research groups. This is clearly seen in the studies and developments discussed in this review. More comparative studies between AI models and training methods moving forward would better characterize the trade-offs and use cases for different training algorithms. Ultimately, this should lead to standardization of AI development and training.

Beyond technical limitations and considerations, there are legal and ethical challenges that will need to be resolved as AI becomes more widely adopted. Clinical data may become commercialized, invoking questions of privacy and ownership of patient data. Legally, difficulties may arise from the black-box nature of current AI algorithms [37]. The complexity of these algorithms and the mechanics of AI training make it extremely difficult for humans to understand the logic behind decisions made by the AI. This makes the importance of trained experts even more necessary in an enhanced AI workflow as the ultimate decision maker.

## 6. Future Outlook

Future directions include scaling AI-based EUS research to further demonstrate the benefit of AI in clinical practice. The first hurdle would be to take the insights gleaned from individual small-scale studies and create an AI tool for EUS that is robust enough to aid endoscopists in various situations and diverse populations. This invariably will require standardization of the technology and operator training.

An important potential contribution AI can provide would be to improve access in more resource limited areas. Improvements in efficiency with the use of AI would help increase the number of procedures endoscopists can perform in a day, contributing to timelier EUS procedures for patients.

Advances in mathematics and computer science create possibilities for future applications to EUS. Such advances include Transformative Noise Reduction, a proposed AI method for image denoising of complex noise patterns [38]. However, it will take time for new developments in the underlying technology to be translated into clinical impact.

Development of AI tools has been focused on established EUS use cases such as diagnosis and biopsy. AI has already been applied to ultrasound elastography [39].

The future of AI in EUS is promising, however most of the studies and their respective AI models thus far have been validated with retrospective data often from single centers. Large scale multi-center prospective trials will be a necessary and important step in the maturation process for AI in EUS. These trials should involve multiple countries and diverse populations to maximize external validity. Diverse training data, specific data sets for specific populations, standardized validation methods, and real time AI model performance monitoring and updates will help minimize biases [40].

## 7. Conclusions

AI is currently undergoing rapid development and is becoming increasingly prevalent across many fields in science and medicine, and the gastroenterology field is no exception. Advanced endoscopists who perform EUS will benefit from developments in using AI in EUS for the detection and diagnosis of neoplasms, improvement of the EUS-guided biopsy process, and training of new operators. While there are many limitations and potential pitfalls of the technology, the future of AI in EUS is bright.

## Figures and Tables

**Table 3:** Role of AI in biopsies via EUS.

Study	Country of Study	AI Model	Area of Interest	Number of Training Images	Number of Test Images	Primary Outcome
Inoue 2014 [17]	Japan	GMM	AI visual tool to aid ROSE	Not reported	Not reported	Qualitatively successful highlighting of tumor cell areas
Hashimoto 2018 [21]	Japan	ANN	Application of ROSE to PDAC	300	150	Sensitivity 80.0%, Specificity 80.0%, Accuracy 80.0%
Kurita 2019 [25]	Japan	ANN	Application of ROSE to Pancreatic Cystic Lesions	85	85	Sensitivity 95.7%, Specificity 91.9%, Accuracy 92.9%
Ishikawa 2022 [22]	Japan	CNN	MOSE in pancreatic diseases	173	173	Sensitivity 90.3%, Specificity 53.5%, Accuracy 84.4%
Kuwahara 2023 [24]	Japan	CNN	Application of ROSE to Pancreatic Cystic Lesions	110	85	Greater Sensitivity, Specificity, and Accuracy compared to Endoscopists
Qin 2023 [27]	China	CNN	EUS-FNA/B diagnosis of PDAC	72	72	Sensitivity 93.1%, Specificity 91.2%, Accuracy 92.0%
Fujii 2024 [26]	Japan	CNN	Application of ROSE to EUS/FNA	3320	830	Sensitivity 87.5%, Specificity 79.7%, Accuracy 83.7%

Abbreviations: GMM (Gaussian mixture model), ANN (artificial neural network), CNN (convolutional neural network), AI (artificial intelligence), ROSE (rapid on-site evaluation), PDAC (pancreatic ductal adenocarcinoma), MOSE (macroscopic on-site evaluation), EUS-FNA/B (endoscopic ultrasound fine-needle aspiration/biopsy).

**Table 4:** Role of AI in endoscopic training with EUS.

Study	Country of Study	AI Model	Area of Interest	Primary Outcome
Zhang 2020 [33]	China	CNN	EUS Training	Trainee accuracy improved from 67.2% to 78.4%
Yao 2021 [30]	China	CNN	AI on training of novice endoscopists	Lower Miss Rates in AI-assisted novices
Bonmati 2022 [35]	UK	CNN	Vocal Input and Labeling During EUS benefiting Training	Successful vocal input tool with accuracy of 76.0%
Tang 2023 [32]	China	CNN	CH-EUS Training	Intersection over Union increase from 0.80 to 0.87

Abbreviations: CNN (convolutional neural network), EUS (endoscopic ultrasound), AI (artificial Intelligence), CH-EUS (contrast-enhanced harmonic EUS).

## Data Availability

No new data were created or analyzed in this review paper. Data sharing is not applicable to this article.
